# The Medical Association

**Published:** 1894-05

**Authors:** 


### *EDITORIAL.*

---

The office of THE JOURNAL is in room 330 Equitable Building.

Address all communications, and make all remittances payable to THE ATLANTA MEDICAL AND SURGICAL JOURNAL, Atlanta, Ga.

Articles for publication should reach this office not later than the 15th instant.

The editors are not responsible for views expressed by contributors.

---

### THE MEDICAL ASSOCIATION.

Elsewhere will be found the stenographer's report of the late meeting of the Association in Atlanta. This meeting was one of the most successful of late years. The program embraced an excellent list of good papers, all of which, unfortunately, could not be read. In his opening address, President Elliott presented an old, but ever important subject, in an able and interesting manner. We are pleased to present it in full in this issue. It will be found to contain wisdom and suggestions emanating from several sources upon the physician's relation to his patient.

The Orator's Address, by Dr. Benedict, of Athens, was one of the most notable papers presented to the Association. In a highly scientific and scholarly manner the speaker discussed the physiology and localization of the central functions in the light of our latest knowledge, with special reference to the therapeutics of suggestion.

There were two features of the Association not down on the program. One was the discussion which arose upon the application of Dr. Don M. Bosworth, of Atlanta. Dr. Bosworth's career had been that of a non-conformist to the laws of ethics, while his father is notoriously irregular. The applicant appeared before the Board of Censors and evinced both a spirit of repentance and a desire to reform. After some remarks and inquiries concerning the present status of Dr. Bosworth he was admitted, one vote dissenting.

The other feature alluded to was a sparring between the two medical colleges in this city, which resulted in a slight disfigurement of both institutions. The Dean of the Southern Medical College had preferred certain charges against the Proctor of the Atlanta Medical College, having reference mainly to the graduation of students after an insufficient term of study. Certain charges of a similar character were offered by the latter gentleman against the former, and the outcome of it all was the following report of the Board of Censors (Dr. W. O'Daniel, Chairman): "The Board of Censors having been engaged in protracted deliberations over charges and counter-charges . . . etc., . . find that both of these gentlemen have, in their zeal to support their colleges, been guilty in their official acts of indiscretions in violation of the spirit of our Code of Ethics. We deem it unnecessary to bring any charges against either of them before this Association."

Dr. Richard Douglas, of Nashville; Dr. H. E. Stafford, of New York, and Dr. Frank Trester Smith, of Chattanooga, were visitors to the Association and were invited to participate in the discussions. Dr. Douglas spoke upon the "Treatment of Surgical Shock," and Dr. Stafford presented a paper on "The Extraction of Clear Lenses."

On Thursday evening a reception was given at the Capital City Club, which was well attended by the members. The magnificent rooms of the club were thrown open and elegant refreshments served to the soft music of the Artillery Band.

Friday at 11 o'clock, the election of officers was taken up as the regular order. This finally resulted as follows: President, Willis F. Westmoreland, Atlanta; First Vice-President, Dr. R. H. Taylor, Griffin; Second Vice-President, Dr. Wm. B. Tate, Tate; Secretary, Dr. Dan. H. Howell, Atlanta; Board of Censors, Drs. W. O'Daniel, G. W. Milligan, C. D. Hurt and F. M. Ridley.

The next meeting of the Association will be held in Savannah.

